# Supplementary figures and images for: Mitochondrial mRNA localization is governed by translation kinetics and spatial transport
Source: PLoS Comput Biol. 2022 Aug 19;18(8):e1010413. doi: 10.1371/journal.pcbi.1010413 (PMC9432724; doi:10.1371/journal.pcbi.1010413)

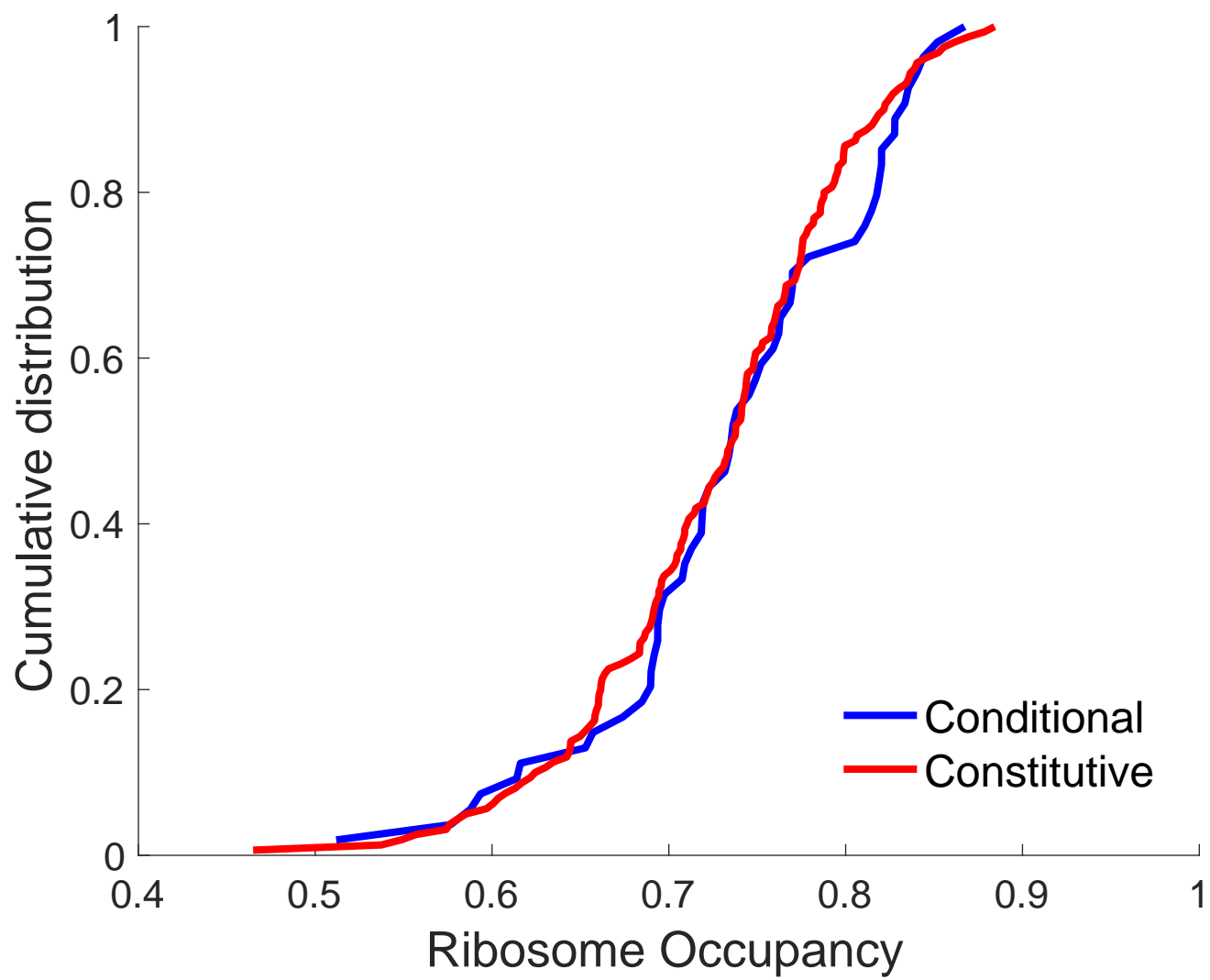

Supplement: S1 Fig — Ribosome occupancy from Arava et al [43]. nconditional = 54 and nconstitutive = 160. These ribosome occupancy values cover a distinct range, in comparison to those of Fig 2A, due to distinct experimental measurement techniques. (PDF) [file pcbi.1010413.s001.pdf]

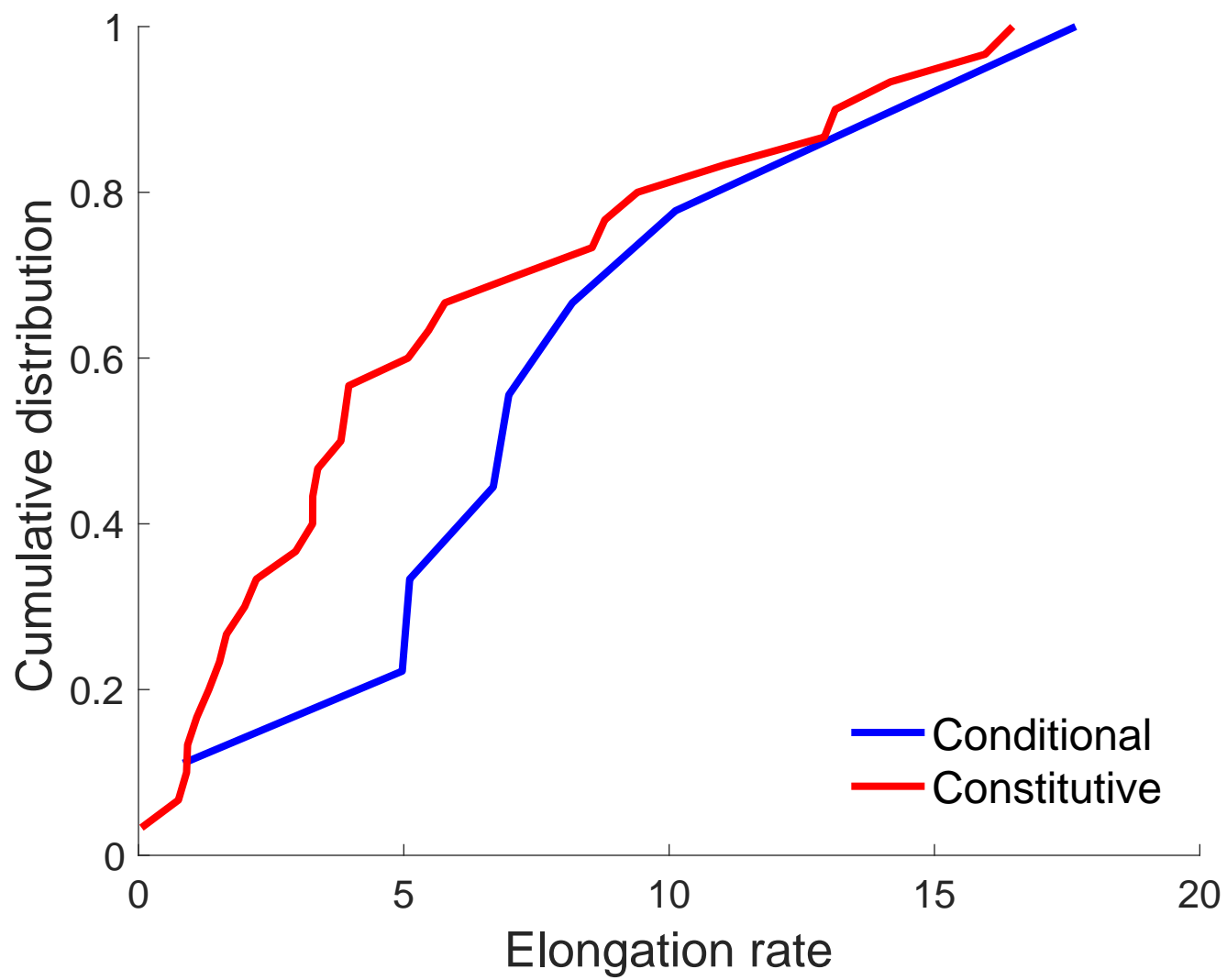

Supplement: S2 Fig — Elongation rates calculated with data from and as described in Riba et al [42], with elongation rate equal to protein synthesis rate divided by ribosome density. nconditional = 9 and nconstitutive = 30. (PDF) [file pcbi.1010413.s002.pdf]

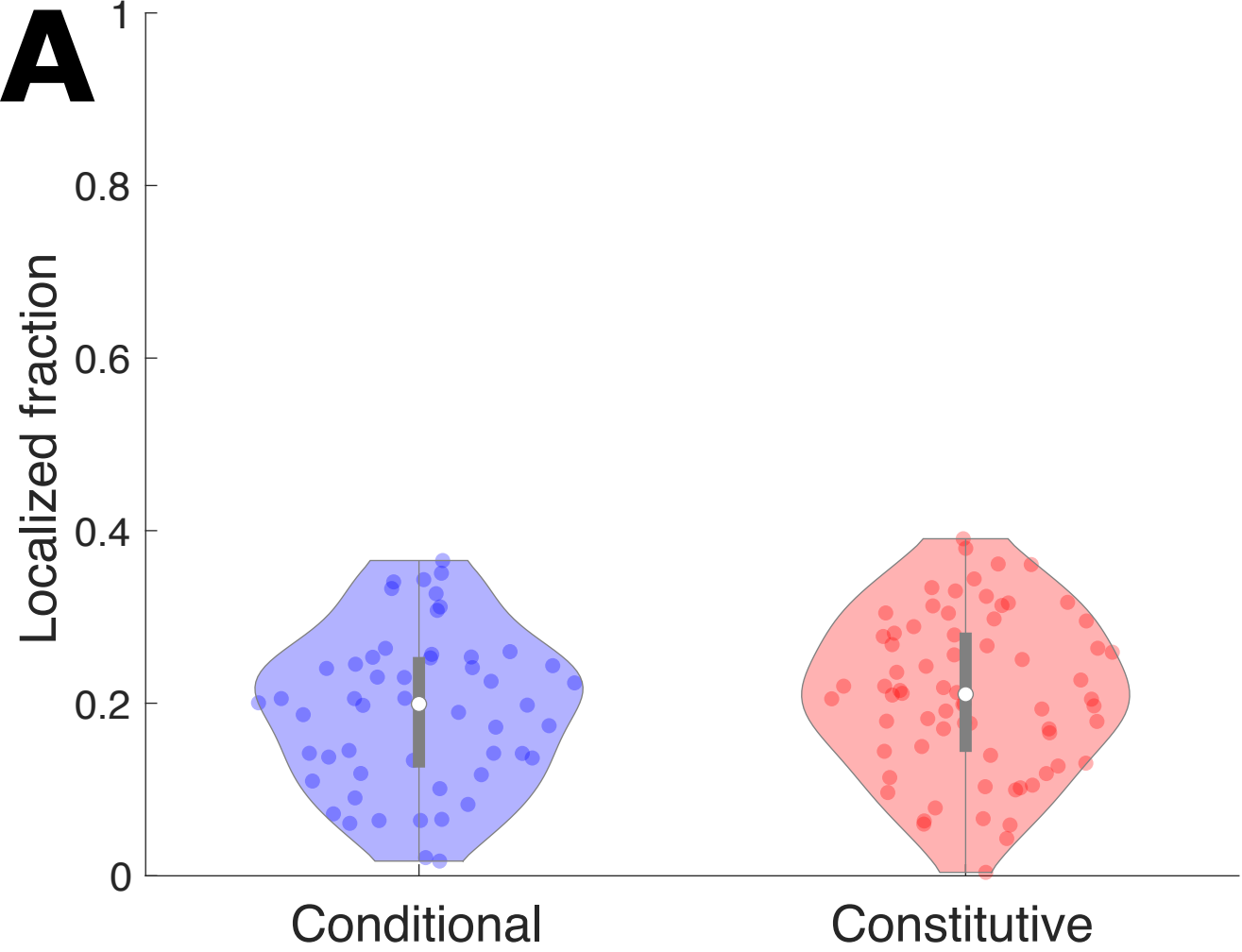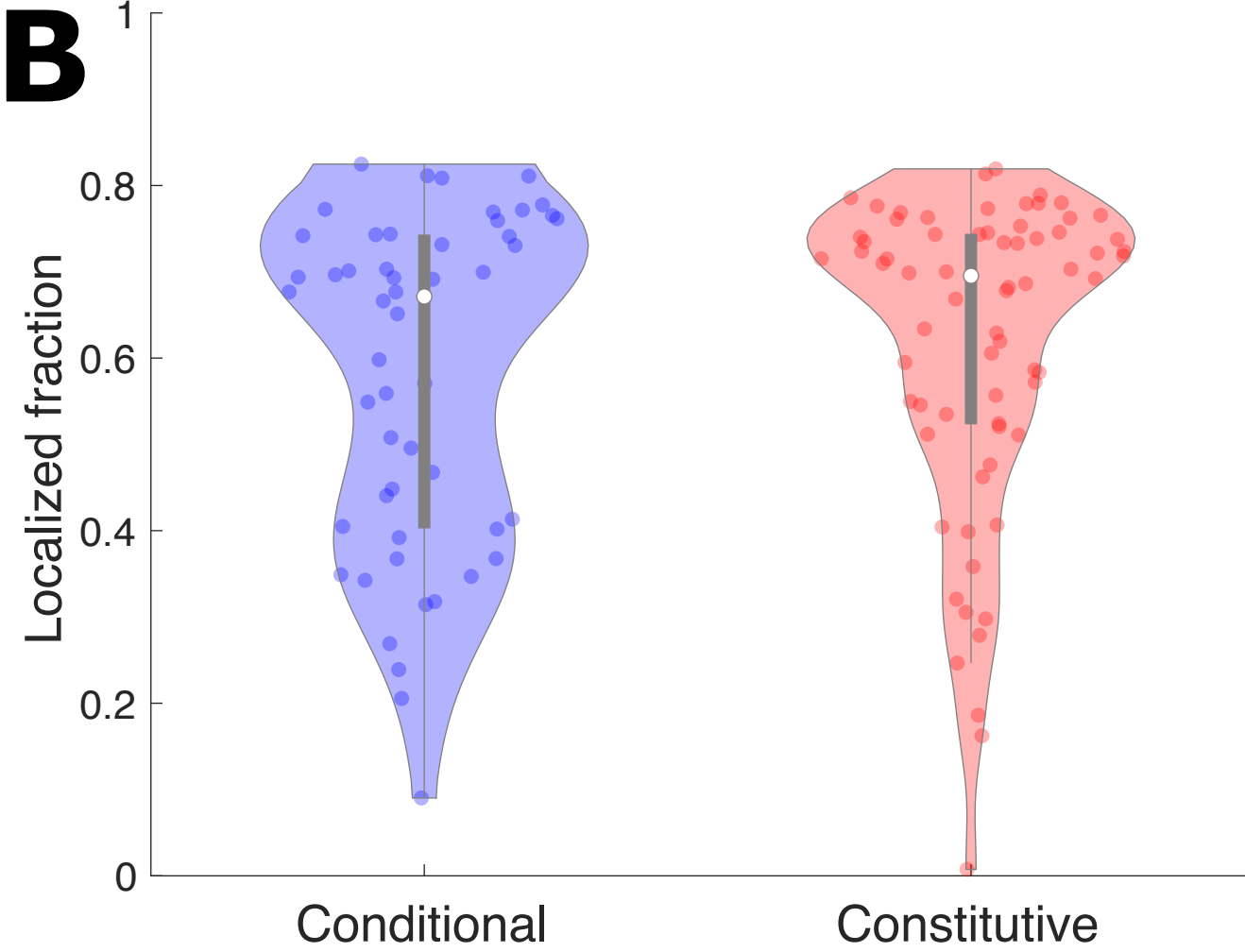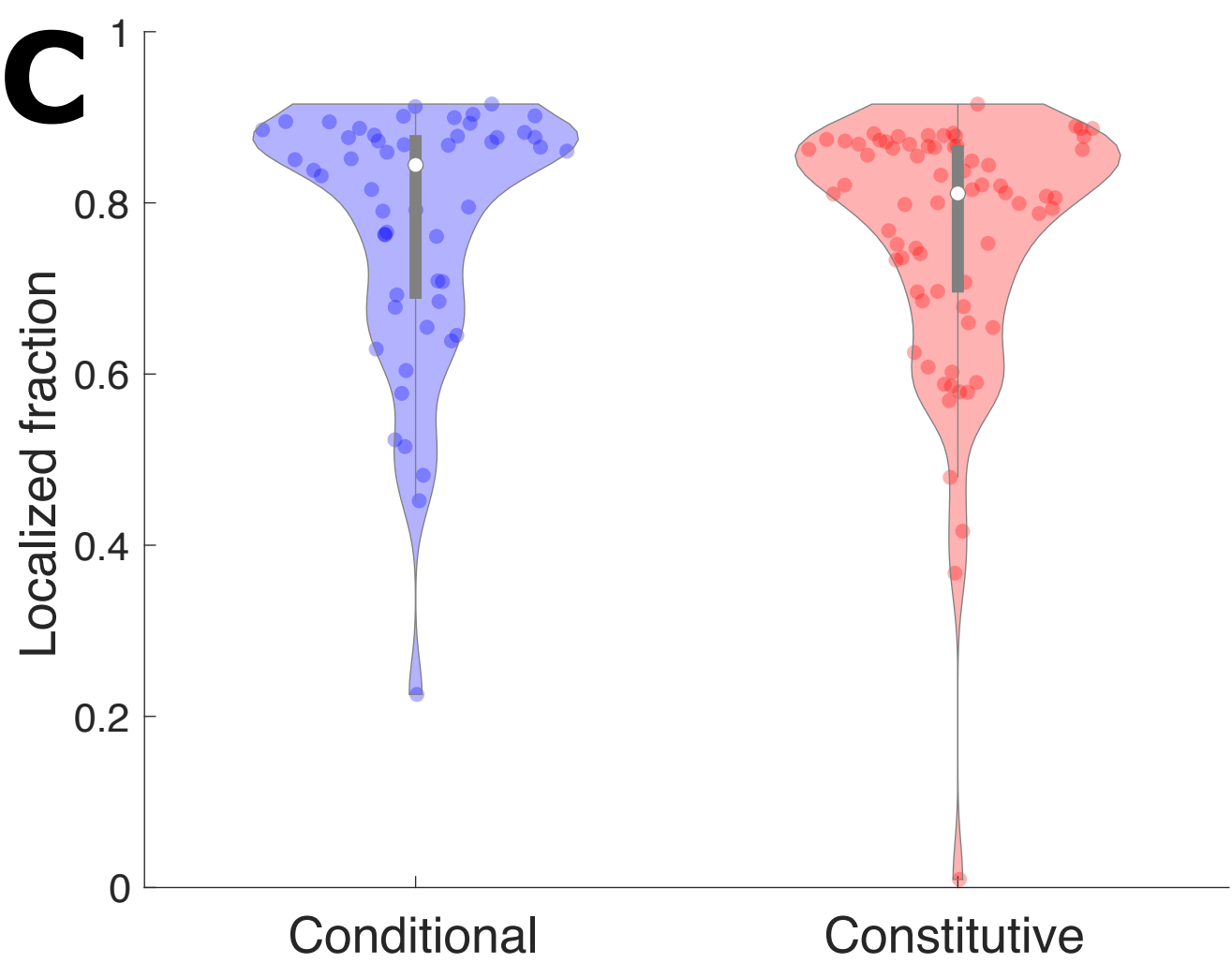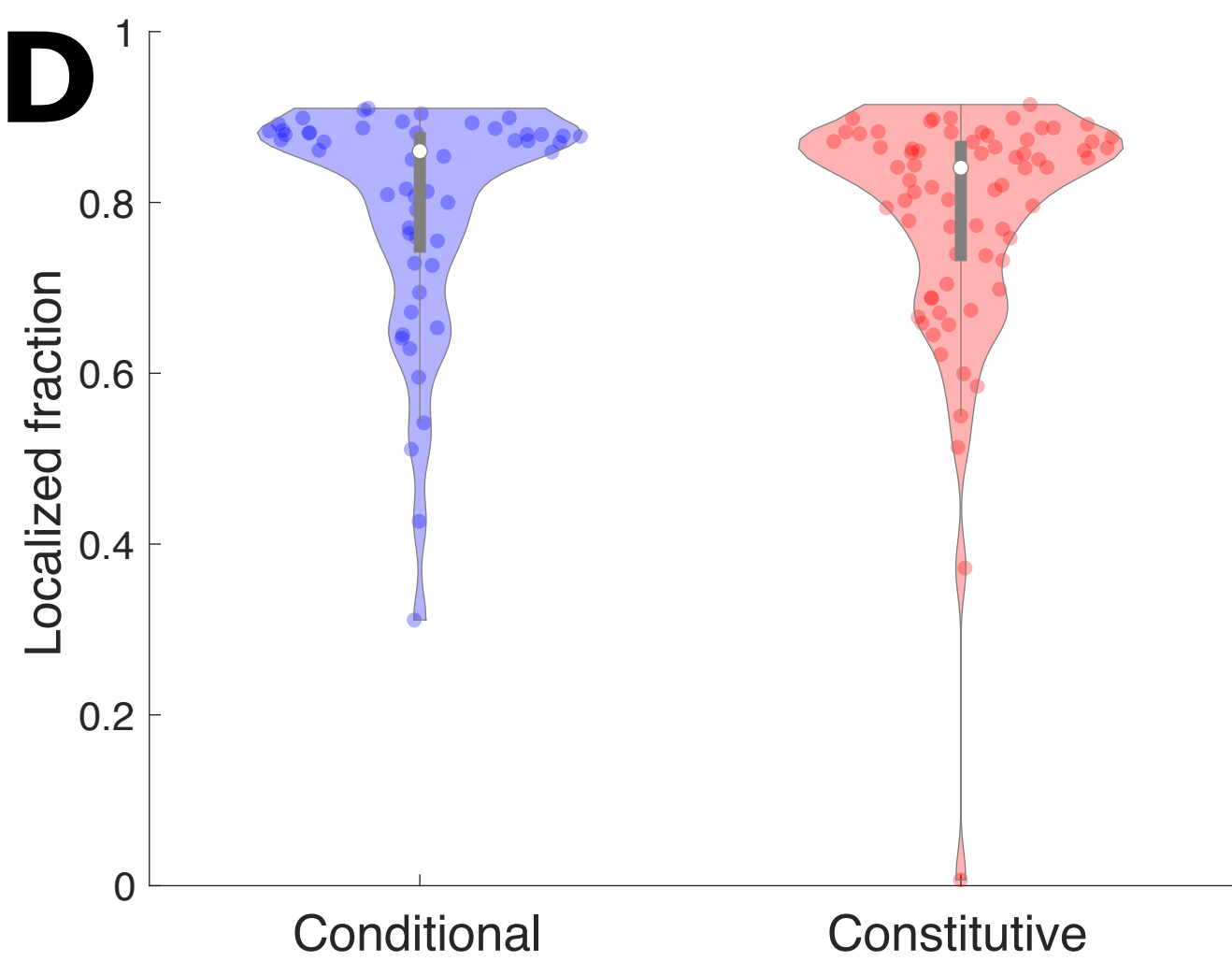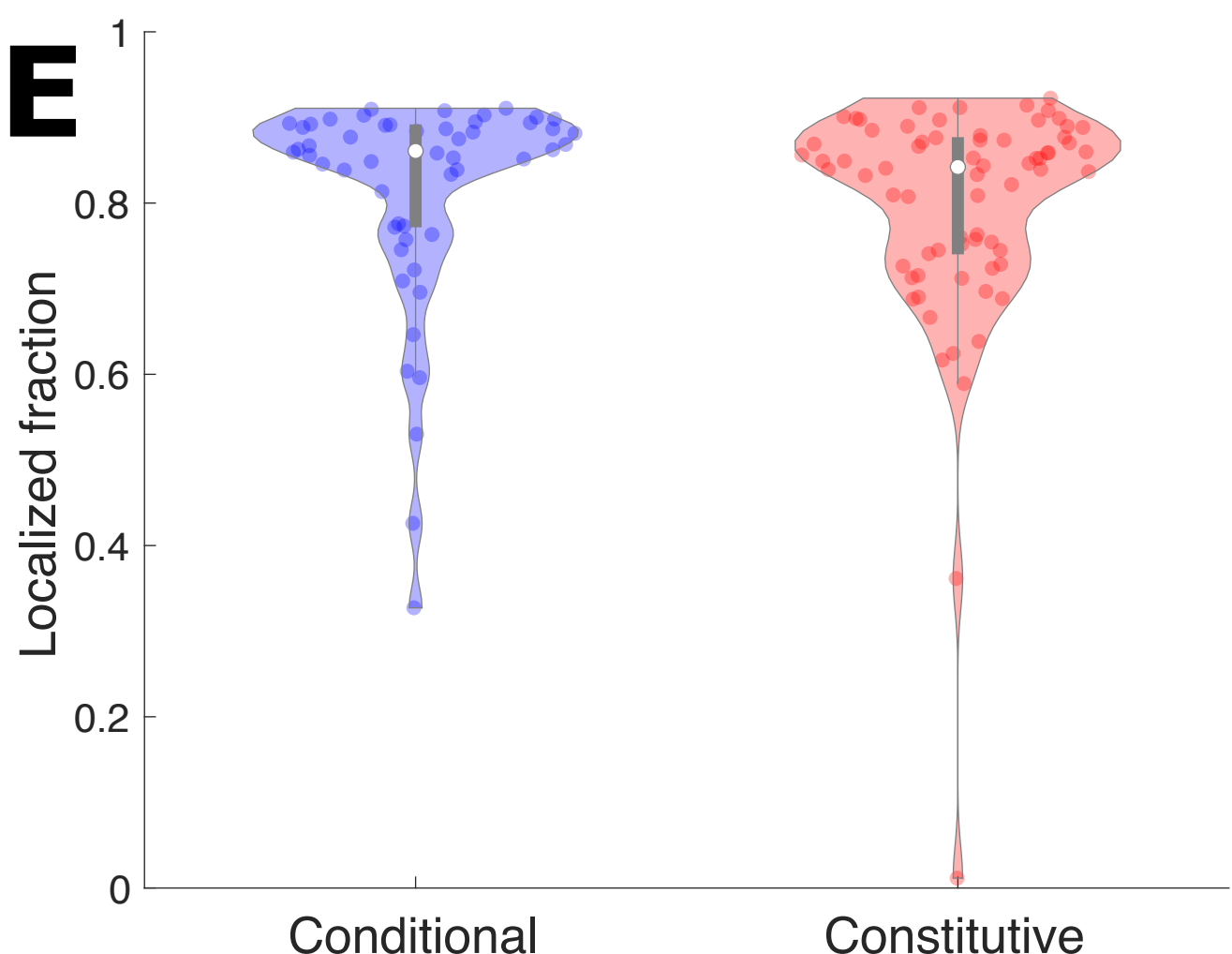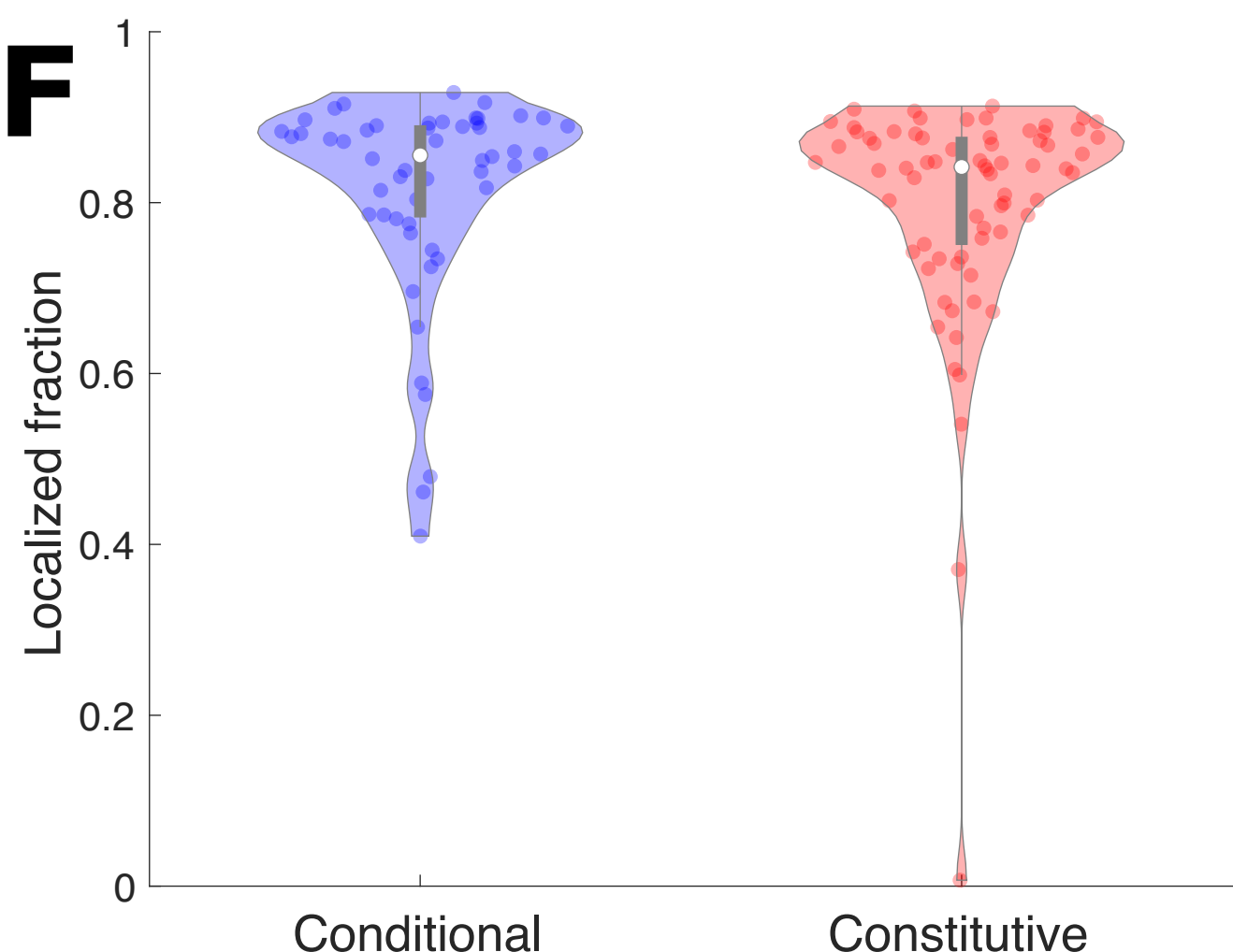

Supplement: S3 Fig — (A) is with mRNA diffusivity D = 0.001 μm2/s, (B) with D = 0.01 μm2/s, (C) with D = 0.1 μm2/s, (D) with D = 0.2 μm2/s, (E) with D = 0.5 μm2/s, and (F) with D = 1 μm2/s. (PDF) [file pcbi.1010413.s003.pdf]

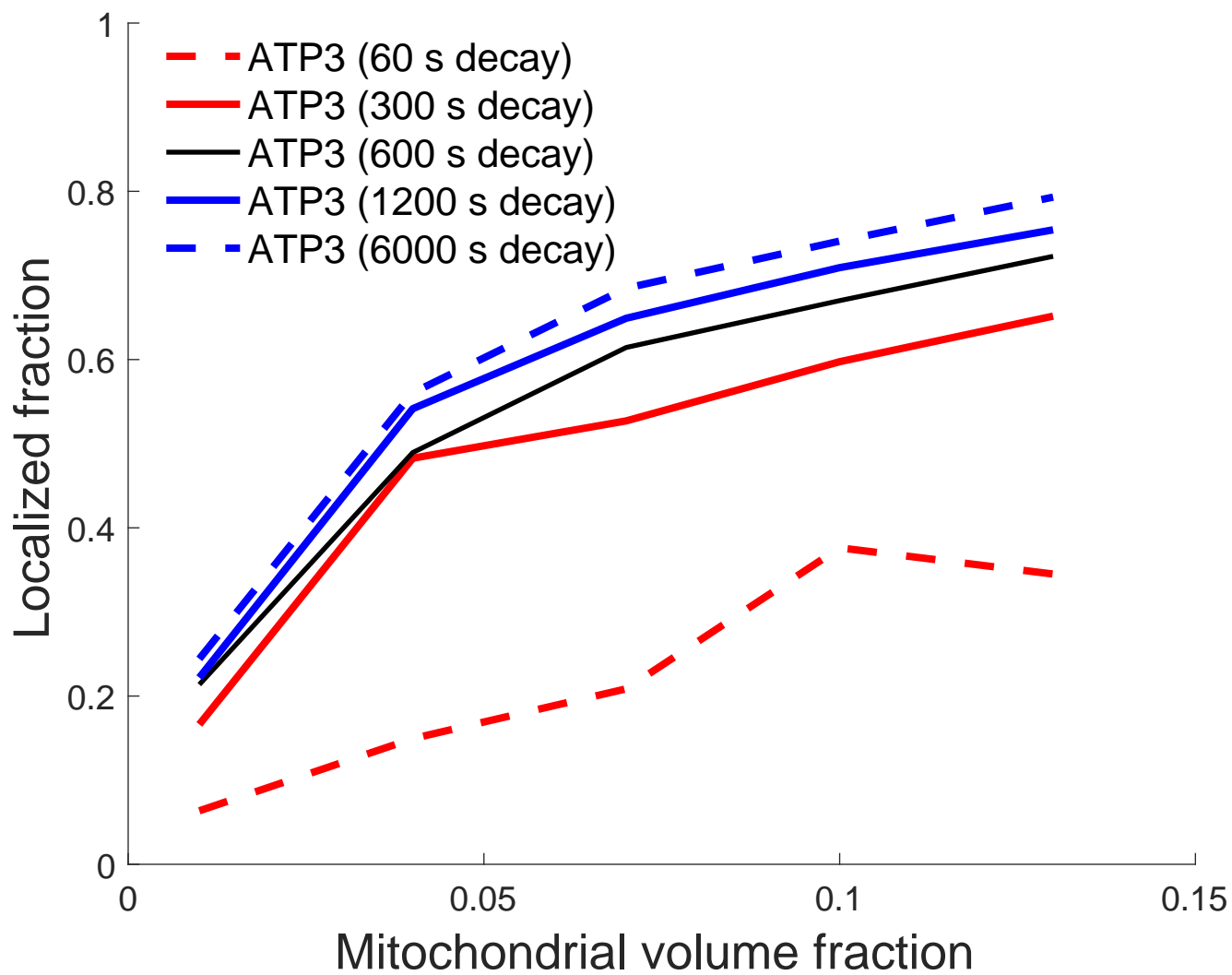

Supplement: S4 Fig — ATP3 mRNA decay time is varied, with the 600 s decay timescale used in other figures. Decay timescale has limited impact unless it is sufficiently short to compete with the timescale for a newly-synthesized mRNA to first gain binding competence. (PDF) [file pcbi.1010413.s004.pdf]
