# Supplementary material for: Mitochondrial mRNA localization is governed by translation kinetics and spatial transport
Source: PLoS Comput Biol. 2022 Aug 19;18(8):e1010413. doi: 10.1371/journal.pcbi.1010413 (PMC9432724; doi:10.1371/journal.pcbi.1010413)
Supplement: Attachment — Submitted filename: 124871_1_rebuttal_2356961_rcwkc8.pdf [file pcbi.1010413.s006.pdf]

Full Revision

Review COMMONS

Manuscript number: RC-2022-01314
Corresponding author(s): Brian Zid and Aidan Brown

[Please use this template only if the submitted manuscript should be considered by the affiliate journal as a full revision in response to the points raised by the reviewers.

If you wish to submit a preliminary revision with a revision plan, please use our "Revision Plan" template. ***It is important to use the appropriate template to clearly inform the editors of your intentions.****]*

1. General Statements [optional]

Dear editors,

We would like to submit our manuscript "Mitochondrial mRNA localization is governed by translation kinetics and spatial transport" for publication in PLoS Computational Biology, after receiving reviews via the Review Commons.

As detailed in our manuscript, we have developed a quantitative model to describe mitochondrial targeting sequence-mediated mRNA localization to mitochondria in yeast. Our work indicates that yeast cells can regulate mRNA localization to mitochondria by controlling mitochondrial size and gene translation kinetics, providing an insight that would be difficult to obtain through experiment alone. This work has specific appeal to biologists and biochemists studying mRNA dynamics, protein synthesis, and mitochondria, and is also of broad interest to researchers in cell and molecular biology.

The reviewers appreciated both the scientific impact of our work and how this work is presented in the manuscript. The reviewers described our manuscript as "well written" and "the research explained in a remarkably clear and focused style which makes it easy to follow", writing that "the analysis is of high quality" and "the conclusions are convincing". Furthermore, the reviewers felt that we used "models with impactful consequences on the understanding of mitochondrial mRNA localization", that "one of the main conclusions of this work, which is that cells might use different translation dynamics to control mRNA localization, is truly exciting", and that this work "should be appreciated by a broad scientific audience."

The reviewers outlined specific constructive suggestions to improve our manuscript. We are grateful to the reviewers for their thoughtful reading and helpful feedback on the manuscript. We have made changes to our manuscript, as recommended by the reviewers. Specific responses to the reviewer comments and the associated manuscript changes are detailed below.

We attach a revised manuscript which addresses the reviewers' points, a pdf that highlights changes in red and blue, and new Supplemental Information.

Sincerely,

Full Revision

Ximena G Arceo
Elena Koslover
Brian Zid
Aidan Brown

2. Point-by-point description of the revisions

Reviewer #1 (Evidence, reproducibility and clarity (Required)):

Unlike other cell organelles, mitochondria contain a small fraction of their genetic information. However, most of the genetic information about mitochondrial proteins is still in the cell's nucleus and the localization of the respective proteins to mitochondria is facilitated by localized translation of their mRNAs. In turn, the mRNA localization to the mitochondria is partly due to the co-translational association, via the mitochondrial target sequence (MTS) of the nascent peptide.

The manuscript "Mitochondrial mRNA localization is governed by translation kinetics and spatial transport" investigates the mechanisms of mRNA transport and attachment to mitochondria. Concerning mitochondria-localized mRNAs, two types of mRNAs have been distinguished before: mRNAs that are always attached to the mitochondrium (called "constitutively binding" by the authors) and mRNAs that become "sticky" only under certain conditions (called "conditionally binding" by the authors). Modeling the corresponding cellular processes biophysically, the authors infer that yeast cells exercise control over the localization of mRNA (and consequently over their metabolism) in two ways: via varying the mitochondrial volume fraction, and via varying the speed of translation elongation. Data from previously published genome-wide measurements of mRNAs that localize constitutively and conditionally via their MTS in budding yeast S. cerevisiae were used to investigate these mechanisms.

The manuscript is very well written and the analysis is of high quality. It starts with an introduction that thoroughly reviews many facets around the conducted research and briefly, but self-consistently, summarizes the current knowledge regarding mitochondrial localization of mRNAs. Next, the consequences of the modeling work (presented in the "methods"-section) are explored in the "Results"-section, which contains meaningful and instructive figures and explanations. The manuscript concludes with a comprehensive evaluation of the consequences of the conducted research. All in all, there are only very few minor changes that could be considered.

Content-wise, we suggest:

The modeling of translation kinetics is pretty coarse-grained, using only an average elongation rate per amino acid. Much work in this field was done using totally antisymmetric exclusion principle (TASEP)-based models (e.g. MacDonald, J.H. Gibbs, A.C. Pipkin: Kinetics of biopolymerization on nucleic acid templates; Duc, Saleem, Song: Theoretical analysis of the distribution of isolated particles in totally asymmetric exclusion processes: Application to mRNA translation rate estimation). Perhaps this work can be mentioned, and furthermore, the consequences of inhomogeneity of elongation rate for different codons and amino acids could

be explored or at least discussed. In particular, this could shed light into the question if ribosome interference and tRNA charging times have any impact on mitochondrial mRNA localization.

Thank you to the reviewer for pointing us to these relevant papers. As suggested, we have added a paragraph to our Discussion that mentions this work and discusses the possible implications of inhomogeneous elongation along mRNA sequences. We find this suggestion (and the similar one made by the other reviewer) to explore inhomogeneous elongation particularly encouraging, because we are in the early stages of actively pursuing such work. We feel that beyond discussion, exploring the consequences of inhomogeneous elongation is beyond the scope of this work because significant further experimental work would be needed to quantify the impact of specific sequences on translation progress.

To our Discussion, we have added the following paragraph.
"In this work our quantitative model assumed uniform ribosome elongation rates along mRNA transcripts. In the presence of ribosome interactions, such dynamics can lead to both uniform and non-uniform ribosome densities and effective elongation rates along the transcript (MacDonald et al., 1968; Duc et al., 2018). With these uniform ribosome elongation rates, previous theoretical results suggest that collisions will be rare (Duc et al., 2018). However, elongation may not be homogeneous along an mRNA transcript, due to factors such as tRNA availability (Varenne et al., 1984), boundaries between protein regions (Thanaraj and Argos, 1996), amino acid charge (Charneski and Hurst, 2013), and short peptide sequences related to ribosome stalling (Sabi and Tuller, 2017). We have found that slow (homogeneous) elongation facilitates mitochondrial mRNA localization, by providing time for MTS maturation, diffusive search, and to maintain binding-competent MTS-mediated mRNA binding to mitochondria. We expect that inhomogeneities in elongation rate along mRNA could either enhance or reduce mitochondrial mRNA localization, controlled by whether slower elongation is in regions that favor longer MTS exposure. For example, a ribosome stall site following full MTS translation could provide more time for MTS maturation and facilitate mitochondrial localization. Future experimental work could identify such stalling sequences and point towards how modeling can improve understanding of sequence impact on localization."

Ribosome occupancy data from Arava used to infer translation parameters. But there are more recent data sets based on ribosome profiling. Any reason for not using the more recent data?

We thank the reviewer for bringing up this important point. Our text describing the origin of data for ribosome occupancy in the inset of Figure 2A lacked a citation to the dataset used, and we agree that more recent ribosome occupancy datasets are more appropriate. For the cumulative distributions of ribosome occupancy shown in the inset of Figure 2A, we used the ribosome occupancy data from Zid and O'Shea from 2014. The Arava data from 2003 was used for the cumulative distributions of Figure S1, to show that the similarity between conditional and constitutive genes in the inset of Figure 2A was present in more than a single dataset.

We have clarified the origin of the ribosome occupancy data in the text.
In the text description of the inset of Figure 2A, we now include a direct citation of Zid and O'Shea from 2014.
"These measurements (Zid and O'Shea, 2014) indicate that conditional and constitutive genes have similar distributions of ribosome occupancy (Fig. 2A, inset; see Fig. S1 for similar

distributions of conditional and constitutive gene ribosome occupancy derived from (Arava et al., 2003))."

We also added a citation of Zid and O'Shea to the caption describing the inset of Figure 2A. "Inset is cumulative distribution of ribosome occupancy (Zid and O'Shea, 2014), showing ribosome occupancy and β have similar distributions. "

To determine the translation parameters in our quantitative model, we applied the datasets of Couvillion et al from 2016 for relative protein per mRNA measurements and Zid and O'Shea from 2014 for ribosome occupancy measurements, combined with individual measurements from Morgenstern et al from 2016 and Riba et al from 2019. How these datasets and measurements are used is described in the Methods subsection "Calculation of translation rates". In addition to the citations in the methods, we have added citations to the briefer description in the Results section.
"Using protein per mRNA and ribosome occupancy data (Couvillion et al., 2016; Morgenstern et al., 2017; Zid and O'Shea, 2014; Riba et al., 2019), we estimated the gene specific initiation rate kinit and elongation rate kelong for 52 conditional and 70 constitutive genes (see Methods)."

The effect of the mitochondrial volume fraction on mRNA localization is investigated with a diffusive model. However, the authors make a two dimensional Ansatz for the cell and mitochondrion while it would seem more natural to assume diffusion in three spatial dimensions, as the cell and mitochondria are both three dimensional objects and diffusion strongly depends on the number of dimensions it occurs in. Why was that Ansatz made and why is it justified?

Our diffusion model is in fact three-dimensional, rather than two dimensional. Specifically, we treat the search process as occurring in a three-dimensional cylinder, whose cross-section is shown in Figure 1D. We have added to Figure 1D to further describe how three-dimensional cylinders represent the mitochondrial proximity in the cell.

In the Results, we now write:
"Specifically, we treat the geometry as a sequence of concentric three-dimensional cylinders, each representing an effective region surrounding a tubule of the mitochondrial network. Figure 1D shows a two-dimensional cross-sectional view of these cylinders. The innermost cylinder represents a mitochondrial tubule…"

We have also clarified the caption of Figure 1D to include:
"Schematic of mRNA diffusion in spatial model, shown in cross-section. The cytoplasmic space is treated as a cylinder centered on a mitochondrial cylinder: the three dimensional volume extends along the cylinder axis (not shown)."

The range of variability in the localized fraction +/- CHX is smaller in the experiment compared to the model (Fig. 4B, C). What could be the rationale?

We agree that the variability in localized fraction from applying CHX is smaller in the experiment (Figure 4C) in comparison to the model (Figure 4B). Our model uses translation parameters

(initiation and elongation rates) that are derived from experimental measurements that are expected to be quite noisy. We expect that this noise in the model parameters will expand the range of localization changes predicted by the model for CHX application.

In l. 417, the authors remark that "constitutively localized mRNAs are on average longer [...] than conditionally localized mRNAs." Yet constitutively localized mRNAs seem to have higher localized fraction than conditionally localized mRNAs. This is somewhat surprising. While it's clear that a higher diffusivity would be compatible with a faster
response time of shorter, conditionally-localized mRNAs, it is not clear how the longer, less diffusive mRNAs would have a higher localization fraction. Perhaps the authors can clarify this point.

The reviewer is correct that experimental measurements show that constitutively-localized genes are, on average, longer than conditionally-localized genes. In our quantitative model, we assume the mRNA of all genes have the same diffusivity. We have used the same diffusivity for different genes because experimental measurements suggest that mRNA length and the number of translating ribosomes on an mRNA do not substantially impact mRNA diffusivity. In our Methods section, we have added citations to papers indicating lack of dependence of mRNA diffusivity on mRNA length.
"Simulated mRNA have a diffusivity of 0.1 $\mu m^2/s$. This diffusivity remains constant across genes and mRNA states, consistent with experimental measurements showing little dependence of mRNA diffusivity on mRNA length (Calderwood et al., 2016) or number of translating ribosomes (Wang et al., 2016)."

We have additionally clarified the part of our Discussion where we explain the distinction of our results from proposals based on differential mRNA diffusion speed.
"Lower occupancy was proposed to drive mRNA localization through increased mRNA mobility of a poorly loaded mRNA (Poulsen et al., 2019), as more mobile mRNA could more quickly find mitochondria when binding competent, increasing the localization of these mRNA. By contrast, our results imply an alternate prediction – that translational kinetics lead to enhanced localization of longer mRNAs, due to the increased number of loaded ribosomes bearing a binding-competent MTS. Indeed, constitutively localized mRNAs are on average longer than conditionally localized mRNAs."

Minor formal changes would be:

Setting the expressions of the fraction in the binding-competent state in l. 118 and the faction of the mRNA-accessible volume in l. 123 in normal math-environments instead of the inline-environment since they are of key importance to the following discussion.

These two equations (now equations (1) and (2)) are set as distinct equations that are now referred to by their equation numbers later in the manuscript.

l. 414 contains the verb "vary" twice

Thank you to the reviewer for pointing out this redundancy, the sentence now reads

Full Revision

"Translation kinetics can widely vary between genes ... "

l. 438 lacks an "h" in the word mitochondria

Thank you to the reviewer for pointing this out, this spelling error has been corrected. The sentence now reads "all mRNA transcripts studied would be highly localized to mitochondria in all conditions."

Reviewer #1 (Significance (Required)):

All in all, this is a strong manuscript that contains solid, simple but meaningful and by no means oversimplified models with impactful consequences on the understanding of mitochondrial mRNA localization. Furthermore, it is likely that the approach applies to other cellular compartments like the ER. The research is explained in a remarkably clear and focussed style which makes it easy to follow and meanwhile succeeds in not omitting any details.

Reviewer #2 (Evidence, reproducibility and clarity (Required)):

Summary:

Arceo et al. have developed a stochastic, quantitative model of mitochondrial targeting sequence (MTS)-mediated mRNA localization to mitochondria in yeast. They use this model to investigate the role of translation- and diffusion kinetics in controlling mitochondrial mRNA localization of conditional as well as constitutional genes.

Most importantly, they find that neither mRNA diffusivity nor ribosome density alone are sufficient to account for the differences in localization that were experimentally observed for the two types of genes. Therefore, they implement an MTS maturation time into their model and find that they can now predict gene specific localization rates. Based on these observations, the authors conclude that yeast cells can regulate the localization of mRNAs to mitochondria through (controlling mitochondrial volume fractions and) differences in translation kinetics, which adjust the exposure time and numbers of mature MTSs that are presented on the mRNP and convey binding-competence.

Major comments:

Overall, the manuscript is well written and the conclusions are convincing. The underlying assumptions of the model make sense, but I have no background in modelling and can therefore only comment on the RNA biology aspects and general comprehensibility of the work.

- The authors calculate gene-specific translation initiation and elongation rates to model localization on different transcript classes. In this context,

(i) They use a single decay rate to estimate trajectory lifetime and this decay rate is such (1 nt / 600 s) that it would take the average yeast mRNA (~ 1400 nt; Smith et al., JCB, 2015) 10 days to be turned over. This is not consistent with physiological decay rates and as a consequence, they are essentially not accounting for mRNA turnover. This should be explained in the Methods.

The reviewer has highlighted a lack of clarity in our model description. The mRNA decay rate in the model is (1/600) inverse seconds per entire mRNA molecule, rather than (1/600) inverse seconds per nucleotide. This leads the typical mRNA lifetime to be 600 seconds. The sentence in the Methods section describing the decay timescale now reads "The mRNA decay rate is set to k_{decay} = 0.0017 s^{-1} per mRNA molecule, such that the typical decay time for an mRNA molecule is 600 s. This decay time is consistent with measured average yeast mRNA decay times ranging from 4.8 minutes (Chan et al., 2018) to 22 minutes (Chia and McLaughlin, 1979)."

(ii) Translation and decay are intrinsically linked and translation machinery also recruits decay enzymes. What is more, decay rates differ greatly for different mRNA transcripts. I cannot judge how feasible this is, but it might benefit the model if variable decay rates (i.e. modelled based on translation efficiency?) could be included.

We appreciate this suggestion from the reviewer. We have added a supplemental figure (Figure S4) to explore how mRNA decay rate can impact mitochondrial localization of mRNA. While longer decay rates have little impact on localization, if the decay rate is sufficiently high, the mRNA will have limited opportunity for translation to initiate and a binding-competent MTS to develop, substantially reducing localization. This analysis does not consider how the mRNA lifetime might be coupled with translational effects (such as ribosome stalling). Accounting for the impact of such more complex decay mechanisms would require substantial expansion of the model and extensive additional experiments to parameterize the coupling effects; we believe this extension would be beyond the scope of this manuscript.

To our Discussion, we have added
"While we have focused on how variation in translational kinetics between genes can impact mitochondrial mRNA localization, there is also significant variation in mRNA decay timescales (Chia and McLaughlin, 1979; Chan et al., 2018). Our model suggests (see Fig. S4) that the mRNA decay timescale has a limited effect on mitochondrial mRNA localization, unless the decay time is sufficiently short to compete with the timescale for a newly-synthesized mRNA to first gain binding competence. We leave specific factors thought to modulate mRNA decay, such as ribosome stalling (Mishima et al., 2022), as a topic of future study."

(iii) Along the same lines: Rare codons as well as specific stalling sequences, are known to slow down translation elongation on many transcripts (and will effectively increase MTS exposure time). Can the authors identify transcripts with such signal sequences (on a global scale, apart from TIM50) and incorporate in their model?

We find this suggestion (and the similar one made by the other reviewer) to explore stalling sequences particularly encouraging, because we are in the early stages of actively pursuing such work. We feel that beyond discussion, exploring the consequences of inhomogeneous

elongation is beyond the scope of this work because significant further experimental work would be needed to quantify the impact of specific sequences on translation progress.

To our Discussion, we have added the following paragraph.
"In this work our quantitative model has applied uniform ribosome elongation rates along mRNA transcripts, which with ribosome interactions can lead to both uniform and non-uniform ribosome densities and effective elongation rates along the transcript (MacDonald et al., 1968; Duc et al., 2018). With these uniform ribosome elongation rates, previous theoretical results suggest that collisions will be rare (Duc et al., 2018). However, elongation may not be homogeneous along an mRNA transcript, due to factors such as tRNA availability (Varenne et al., 1984), boundaries between protein regions (Thanaraj and Argos, 1996), amino acid charge (Charneski and Hurst, 2013), and short peptide sequences related to ribosome stalling (Sabi and Tuller, 2017). We have found that slow (homogeneous) elongation facilitates mitochondrial mRNA localization, by providing time for MTS maturation, diffusive search, and maintains a binding-competent MTS-mediated mRNA binding to mitochondria. We expect that inhomogeneities in elongation rate along mRNA could either enhance or reduce mitochondrial mRNA localization, controlled by whether slower elongation is in regions that favor longer MTS exposure. For example, a ribosome stall site after the MTS is fully translated could provide more time for MTS maturation and facilitate mitochondrial localization. Future experimental work could identify such stalling sequences and point towards how modeling can improve understanding of sequence impact on localization."

- Reduced mature MTS exposure time is presented as one of the determining factors that regulate mitochondrial localization of conditionally localized transcripts. For my background, the underlying mechanisms that determine MTS maturation are insufficiently explained. I understand how chaperone recruitment can contribute to MTS maturation. However, it is not obvious to me how receptor binding would account for such long maturation times as the 40 s used here (Fig. 3, 4). I would appreciate if the authors could elaborate and possibly point to directions that their model could be used to study those.

We agree with the reviewer that the diffusive search time for a chaperone to find a newly-synthesized MTS would be very short (a small fraction of the proposed 40-second MTS maturation time), and we expect that this maturation period is largely controlled by chaperone and co-chaperone interaction timescales. There is a wide range of timescales for newly-synthesized (or misfolded) proteins to productively interact with a chaperone, and the literature provides examples of timescales comparable to 40 seconds, which we now cite.
To our Discussion, we have added
"While the diffusive search for a newly-synthesized MTS by chaperones is expected be very fast (<< 40 s), chaperone- and co-chaperone-mediated folding can occur on timescales comparable to 40 seconds, including approximately tens of seconds in bacterial homologs (Sharma et al., 2010; Banecki and Zylicz, 1996) and > 100 seconds for human chaperone-mediated folding (Wu et al., 2020)."

We feel that modeling chaperone facilitation of MTS folding, to determine the timescale of this process, is very distinct from the topics covered in our manuscript, and thus beyond the scope of this work.

- One of the two main conclusions (at least according to the abstract) from the work is that yeast cells modulate mitochondrial volume fractions to regulate mRNA localization to mitochondria. This is a fact, not a novel finding. The other main conclusion, which is that cells use different translation dynamics to control mRNA localization, is intriguing and deserves more attention. It would be great if the authors could suggest/discuss an experimental approach (i.e. a single mRNA imaging experiment quantifying mitochondrial co-localization and translation kinetics of different reporter constructs) to test this hypothesis.

We appreciate the reviewer raising the point that yeast cells modulate mitochondrial volume fraction to regulate mitochondrial mRNA localization. While we previously showed this relationship between mitochondrial volume fraction and localization, we used experimental techniques (mutations, nutrient sources) that changed many other factors beyond mitochondrial volume fraction. In this work we have used a quantitative model, lacking those extraneous factors, to demonstrate that a change to mitochondrial volume fraction alone can lead to a change in mitochondrial mRNA localization. This work supports our interpretation of those previous experimental results.

To our Discussion we have added the sentence
"Previous experimental work suggested that changing mitochondrial volume fraction could control mitochondrial mRNA localization (Tsuboi et al., 2020) --- our quantitative modeling work provides further support for this mechanism of regulating mRNA localization."

The reviewer also requests a discussion of an experimental approach to test how cells use translational dynamics to control mRNA localization. With the advent of combined mRNA imaging and live translational imaging it would be interesting to directly measure translation in live cells to correlate localization with a time delay. Unfortunately there are currently no published live translational imaging studies in yeast, and thus such a measurement would require the development of the technique in yeast.
To our Discussion, we have added
"Experimentally testing our proposal for translation-controlled localization would involve using combined mRNA and live translational imaging (as yet undeveloped in yeast), to directly measure translation and correlate localization with a time delay, presenting a fruitful pathway for future study."

Minor comments:

- Figure 1: X axis labels between panel E and F are not consistent. Inset in panel F is mainly and first discussed in text. Please do not show data as tiny inset but as separate panel.

We have changed the axis label of Figure 1E to match the axis label of Figure 1G (previously Figure 1F). The inset of the old Figure 1F is now the new Figure 1F, and the old Figure 1F is now the new Figure 1G. We have adjusted the Figure 1 caption and the text description of Figure 1 to match these changes.

Elongation rates of 250 aa per second are not physiological. In mammalian cells elongation has been quantified to proceed between 1 and app. 20 aa per second (Wang et al, 2016; Wu et al., 2016; Yan et al., 2016; Morisaki et al., 2016).

The reviewer is correct that the elongation rates of 50/s and 250/s too large to be physiological. These large values have been deliberately selected to probe the nonequilibrium behavior of the quantitative model to test the prediction of the simpler four-state model, rather than represent physiological behavior.
To the text in the Results section discussing Figure 1F, we have added the following sentence. "We include unphysiologically high elongation rates to compare to the expected behavior from the 4-state model."

Panel E: elongation rate range does not match Fig 1F nor median in Fig 3A.

The reviewer is correct that the elongation rate parameter range of Figure 1E does not match the elongation rates of Figure 1F or the median in Figure 3A. In Figure 1E, we aimed to show that the physiological range of translation parameters can produce a wide range of both MTSs per mRNA and mRNA binding competence for mitochondria.
We have expanded the description of Figure 1E in the text.
"By exploring the physiological range of translation parameters, many orders of magnitude of the mean number of translated MTSs per mRNA (β, see Eq. 5) are covered, which also covers the full range of mRNA binding competence (Fig.1E). We find that, for any set of physiological translation parameters, the number of binding-competent MTS sequences (β) is predictive of the fraction of time (f_s) that each mRNA spends in the binding competent state (Fig.1E)."

- Figure 2A and S1: Please explain how ribosome occupancy is defined here and why it is so different between figures

We have inserted a citation for Zid 2014, to distinguish that the ribosome occupancy measurements in Figure 2A (Zid and O'Shea) and Figure S1 (Arava et al) come from two different techniques. Zid and O'Shea used ribosome profiling to obtain a relative, rather than absolute measurement. While Arava used a technique where they fractioned mRNAs based on the absolute number of ribosomes loaded across 14 fractions of a sucrose gradient, and measured the relative amount of mRNA in each fraction by microarray. So while ribosome occupancy in each paper was calculated in a very distinct manner, the comparison between conditional and constitutively localized mRNAs shows a very similar trend without significant differences in ribosome occupancy between these two classes of mRNAs with either measurement of ribosome occupancy.

To the caption of Figure S1, we have added
"These ribosome occupancy values cover a distinct range, in comparison to those of Fig. 2A, due to distinct experimental measurement techniques."

- Figure 2C: please show experimental data along with model prediction (in the same graph) so that conclusion becomes immediately apparent from figure not just main text. Label clearly (in figure) when experimental and when model data is shown (maybe by using consistent color scheme?)

Full Revision

We have added experimental data to Figure 2C. Throughout the manuscript, we have kept a consistent color scheme for data for mitochondrial localization for ATP3, TIM50, conditional, and constitutive mRNA, whether from model or experimental data. We have applied distinct line types (e.g. solid for model vs. dot-dashed with circles for experimental).

- Figure 4B and C: clearly indicate in figure which are experimental and which are modelled data

In Figures 4B and 4C, we have clarified which data is experimental and which is modeled by adding to the labels for each violin plot. Violin plot labels for model data now read "Model Conditional" or "Model Constitutive" and labels for experimental data now read "Expt Conditional" or "Expt Constitutive".

- Figure 4D: show experimental vs. model data in same graph (at same axis scaling) for comparability

We have added the experimental data, previously in the inset of Figure 4D, to the main part of Figure 4D.

- Line 305: "constitutive" mRNA

Thank you to the reviewer for pointing out this redundancy, the sentence now reads "Figure 3C shows how the localization for the prototypical conditional and constitutive mRNA varies with the maturation time."

- Line 334: "other changes, such as diffusivity, are unable to separate the two gene groups" - what other changes? The authors only show diffusivity (Fig S3).

Thank you to the reviewer for pointing this out. We have revised this sentence to only refer to diffusivity changes.
"While introduction of this maturation time distinguishes the mitochondrial localization of conditional and constitutive gene groups (Fig. 4A vs Fig. 2B), changes to diffusivity are unable to separate the two gene groups (Fig. S3)."

- Line 403-405: maybe useful to argue against lower ribosome occupancies as drivers of nascent chain complex mobilities: Wang at el, Cell, 2016; single translation site imaging experiments indicating that ribosome occupancy is not the main determinant of mRNP mobility.

We thank the reviewer for the direction to this paper, which indeed indicates that ribosome occupancy has limited impact on mRNA diffusivity.
We now cite this paper in our Methods section.
"Simulated mRNA have a diffusivity of $0.1 \mu m^2/s$. This diffusivity remains constant across genes and mRNA states, consistent with experimental measurements showing little dependence of mRNA diffusivity on mRNA length (Calderwood et al., 2016) or number of translating ribosomes (Wang et al., 2016)."

- Line 601-607: include experimental references to explain how measures (25 nm vs 250 nm) were determined/selected.

The reviewer raises a valuable point, as it is important to motivate these lengthscales used in the model.
Microscopy with visible light has a lateral resolution limit of approximately 250 nm, often known as the Abbe limit. Accordingly, we assume that mRNA within 250 nm of mitochondria will be measured as adjacent to mitochondria. To the Methods section, we now include a short explanation and a citation.

Unlike the 250-nm diffraction limit, there is no widely-used reaction range for mRNA binding to intracellular substrates, nor a measurement of the required proximity for an MTS-bearing mRNA to bind to mitochondria. We estimate the 25-nm distance for mRNA binding to mitochondria from the following contributions:

- The yeast ribosome is 25 - 28 nm in diameter, or 13 - 14 nm in radius.
- Yeast MTSs have a length of up to 70 amino acids, with 20 estimated yeast MTS lengths having a mean of 31 amino acids. The MTS forms an amphipathic helix (an alpha helix), which has a pitch of 0.54 nm and 3.6 amino acids per turn, so the 31 amino acids will be approximately 5 nm long
- The MTS will be attached to the ribosome/mRNA by other peptide regions, expected to typically be a few nanometers in length

So overall we estimate a 25 nm range for an MTS-bearing mRNA to bind to mitochondria.

To our methods, we have added this reasoning and accompanying citations.
"We estimate the 25-nm binding distance by combining several contributions. The yeast ribosome has a radius of 13 - 14 nm (Verschoor et al, 1998). The MTS region, up to 70 amino acids long, forms an amphipathic helix (Bacman et al., 2020) a form of alpha helix. With an alpha helical pitch of 0.54 nm and 3.6 amino acids per turn, a 31 amino acid MTS (the mean of 20 yeast MTS lengths (Dong et al., 2021)) is approximately 5 nm in length. An additional few nanometers of other peptide regions bridging the MTS to the ribosome provides an estimate of 25 nm for the range of an MTS-bearing mRNA to bind mitochondria. The 250-nm imaging distance is based on the Abbe limit to resolution with visible light (Georgiades et al., 2016)."

Reviewer #2 (Significance (Required)):

My field of expertise is the development of single mRNA imaging methods to quantify translation/decay dynamics in living mammalians systems. Thus, I cannot judge the significance of this work with respect to the modelling that is presented here.
However, I do appreciate that one of the main conclusions of this work, which is that cells might use different translation dynamics to control mRNA localization, is truly exciting and could be applied to other types of transcripts (this is exactly what SRP does for ER-targeted mRNAs) as well. Because mechanisms that regulate translation in a transcript-specific manner and in different subcellular localizations have only been described for a handful of cases, I think that this observation is worth following up on and should be appreciated by a broad scientific audience.
